# Increased Expression of LIPC Is Associated With the Clinicopathological Features and Development of Head and Neck Squamous Cell Carcinoma

**DOI:** 10.7759/cureus.50202

**Published:** 2023-12-08

**Authors:** Sahith Putluru, Chandra Pandi, Balachander Kannan, Vijayashree J Priyadharsini, Paramasivam Arumugam

**Affiliations:** 1 Molecular Biology Lab, Saveetha Medical College and Hospital, Saveetha Institute of Medical and Technical Sciences, Saveetha University, Chennai, IND; 2 Centre for Cellular and Molecular Research, Saveetha Dental College and Hospitals, Saveetha Institute of Medical and Technical Sciences, Saveetha University, Chennai, IND

**Keywords:** molecular biomarker, cancer prognosis, novel gene discovery, cancer mortality, oral health care

## Abstract

Introduction

Lipase C hepatic type (LIPC) is a member of the lipase family and plays a role in tumor development. However, its specific role in head and neck squamous cell carcinoma (HNSCC) is not well understood.

Objective

This study aims to investigate LIPC gene expression in HNSCC and elucidate its potential role in the context of the disease.

Methods

LIPC expression was analyzed using the Cancer Genome Atlas-HNSCC (TCGA-HNSCC) dataset. Real-time polymerase chain reaction (qPCR) was used to validate LIPC expression in oral squamous cell carcinoma (OSCC) tissue samples, which is the most common type of HNSCC. The LIPC was assessed to find out if there is a link with HNSCC clinicopathological features, prognosis, and tumor infiltration. Functional pathways associated with the LIPC network were also examined.

Results

LIPC expression was found to be elevated in both HNSCC and OSCC tissues. The heightened expression of LIPC correlated with various clinicopathological features and influenced the prognosis of HNSCC patients. The LIPC gene demonstrated connections with several oncogenic genes and proteins, participating in lipid catabolic processes and other pathways. These findings suggest that LIPC expression may play a role in the pathogenesis of HNSCC.

Conclusion

Our study affirms that LIPC expression is linked to the development of HNSCC, suggesting its potential utility as a biomarker or therapeutic target for the disease. However, further functional studies are imperative to validate and expand upon these findings.

## Introduction

Head and neck squamous cell carcinoma (HNSCC), comprising malignancies affecting the oral cavity, oropharynx, and larynx/hypopharynx, stands as the sixth most prevalent cancer globally. In 2018, an estimated 700,000 new cases emerged, bearing a grim prognosis, with 350,000 expected fatalities [1]. Over the last 50 years, transformative advancements in medical, radiation oncology, and surgical techniques have significantly altered the landscape of head and neck cancer treatment. Recent whole genome sequencing has deepened our understanding of HNSCC's molecular pathogenesis [2], unveiling its heterogeneity in terms of anatomic subsites, risk factors, and molecular pathologies, thus complicating investigative and therapeutic efforts.

Traditional risk factors like smoking and drinking independently and synergistically increase the relative risk of HNSCC, with dosage responsiveness [3]. Chronic alcohol and tobacco use are implicated in inducing broad molecular changes in healthy epithelia. Dysregulation of various oncogenes and tumor suppressor genes, akin to other solid tumors, underscores the molecular disruption characteristic of HNSCC [4]. High-risk human papillomavirus (HPV) infections now contribute significantly to oropharyngeal tumors in the Western population [5].

Recent findings indicate that HPV infection may accommodate TP53 mutations, impacting TP53 expression, and highlight multiple alterations linked to the tumor-suppressive effect of TGF-β downregulation in HNSCC [6]. The Cancer Genome Atlas (TCGA) identifies key oncogenes (e.g., HRAS, PIK3CA) and tumor suppressor genes (e.g., CDKN2A, KMT2D, FBXW7, TP53) involved in HNSCC [2,7]. Precision medicine in HNSCC management benefits from identifying altered gene expression signatures, unraveling essential biological pathways, and enhancing our understanding of molecular mechanisms [8-12]. Hence, comprehending the molecular pathways governing oral squamous cell carcinoma (OSCC) proliferation and metastasis is crucial for devising novel therapeutic strategies.

The lipase C hepatic type (LIPC) gene, situated on human chromosome 15, is a member of the lipase family. Spanning 35 kb with nine exons and eight introns, it codes for a 65-kD glycoprotein. Anomalous LIPC expression is associated with metabolic and circulatory system disorders, playing a pivotal role in lipoprotein metabolism [13,14]. Dysfunctional lipid metabolism is prevalent in cancer cells, influencing their biological behavior [15]. Studies propose LIPC's involvement in malignancies; for instance, low LIPC expression correlates with improved prognosis in platinum-based chemotherapy for non-small cell lung cancer [16]. In a mouse model of colon cancer, high LIPC expression facilitates liver metastasis [17]. However, the association between LIPC and HNSCC remains unexplored. This study delves into the relationship between LIPC expression and HNSCC, examining the TCGA-HNSC dataset based on clinicopathological features, prognosis, and tumor infiltration. Additionally, LIPC expression is validated in OSCC, a prominent HNSCC subgroup. Bioinformatic tools are employed to elucidate the LIPC functional pathway through its network of genes and proteins.

## Materials and methods

TCGA-HNSC dataset analysis

To scrutinize LIPC mRNA expression in HNSCC (n=520) and normal tissues (n=44), the UALCAN (The University of ALabama at Birmingham CANcer data analysis portal) database [18] within the TCGA dataset was employed. This database offers users the capability to evaluate protein-coding gene expression and its impact on patient survival across 33 types of cancer, leveraging data from the TCGA project. The UALCAN platform facilitated the examination of the association between LIPC and clinicopathological features of HNSCC as well.

Patient enrolment and sample collection

The study was conducted at Saveetha Dental College and Hospitals, Chennai, India. Between November 2022 and April 2023, we enrolled 40 patients diagnosed with OSCC for the purpose of obtaining tumor and adjacent non-tumor tissue samples during surgical procedures. Both OSCC primary tumors and adjacent non-tumor tissues were meticulously collected from these patients and subsequently confirmed through histopathological examination. Following the confirmation of samples as tumors and adjacent tissues, they were promptly preserved at -80 °C until further processing. Detailed clinicopathological information about the patients can be found in Table 1. This research received ethical approval from the Institutional Human Ethical Committee, Saveetha Dental College and Hospitals, Chennai, India, (IHEC/SDC/FACULTY/20/PERIO/01) and strictly adhered to the ethical principles outlined in the Declaration of Helsinki. Furthermore, all participating patients provided informed consent by signing a consent form

**Table 1:** Clinical features of patients with oral squamous cell carcinoma RMT: retromolar trigone; GBS: gingivobuccal

S.No.	Variable	Category	No. of patients (%)
1	Gender	Male	32 (80)
		Female	8 (20)
2	Age	< 50 years	17 (42.5)
		> 51 years	23 (57.5)
3	Grade	Well-differentiated	23 (57.5)
		Moderately differentiated	15 (37.5)
		Poorly differentiated	2 (5)
4	Site	Buccal	12 (30)
		Tongue	9 (22.5)
		Other (RMT,GBS,Maxilla, Mandible)	19 (47.5)
5	Stage	1	5 (12.5)
		2	8 (20)
		3	8 (20)
		4	19 (47.5)
6	Laterality	Left	15 (37.5)
		Right	25 (62.5)
7	Pattern of Invasion	Cohesive	24 (60)
		Non-cohesive	16 (40)
8	Invasion to adjacent sites	Present	15 (37.5)
		Absent	25 (62.5)

RNA extraction and complementary DNA (cDNA) synthesis

Total RNA extraction from both OSCC tumors and adjacent non-tumor tissues was performed using TRIzol reagent (Invitrogen, Thermo Fisher Scientific, Waltham, Massachusetts, USA), following the manufacturer’s instructions. The isolated RNA was quantified using NanoDrop One (Thermo Fisher Scientific, Waltham, Massachusetts, United States). Subsequently, a first-strand cDNA synthesis kit (Takara, Tokyo, Japan) was employed to convert the RNA into cDNA.

Real-time polymerase chain reaction (qPCR) analysis

cDNA, synthesized from the RNA, served as the template for gene expression analysis. Primers, designed using Primer3web (https://primer3.ut.ee/) and ordered from Eurofins (Bangalore, India), are detailed in Table 2. The PCR reaction volume was set at 20 µl, comprising 10 µl 2X SYBR master mix, 50 µL of forward and reverse primers, 2µl of cDNA template, and double-distilled water (DDH2O). qPCR was conducted on a Bio-Rad CFX Opus 96 (Bio-Rad Laboratories, Inc., Hercules, California, United States) with an initial denaturation at 95ºC for three minutes, followed by 40 cycles of denaturation at 95ºC for 10 seconds, annealing at 58 °C for 30 seconds, and utilizing GAPDH as a reference gene. The gene expression results were analyzed using Bio-Rad CFX Maestro 1.0, Version 4.0.2325.0418, software (Bio-Rad Laboratories, Inc., Hercules, California, United States).

**Table 2:** Primer sequence for qPCR qPCR: real-time polymerase chain reaction

Gene	Forward Primer	Reverse primer
LIPC	5’- CACACGTGTCAGGATTTGCC-3’	5’- AGAAAGACGATTGCTGGGGG-3’
GAPDH	5’-TCCAAAATCAAGTGGGGCGA-3’	5’-TGATGACCCTTTTGGCTCCC-3’

In silico functional analysis

In our investigation, the TIMER (Tumor IMmune Estimation Resource) database [19], specifically TIMER2.0, was employed to analyze immune infiltrates across diverse cancer types. Our focus was on utilizing TIMER2.0 to explore the correlation between LIPC expression and immune cell infiltration in TCGA-based HNSCC samples. For the analysis of protein-protein interactions involving LIPC, two platforms were utilized: GeneMANIA [20] and STRING (Search Tool for the Retrieval of Interacting Genes/Proteins) [21]. GeneMANIA utilizes an extensive database of functional association data to identify genes linked to a set of input genes, considering factors such as protein and genetic relationships, pathways, co-expression, colocalization, and protein domain similarity. STRING, on the other hand, is employed for comprehensive protein interaction network construction. Furthermore, Metascape [22] was employed as an additional resource for the functional enrichment study. Interacting genes and proteins obtained from the GeneMANIA and STRING databases were integrated into the Metascape analysis. Metascape combines various public databases, facilitating a comprehensive examination of gene functions, biological processes, and pathways.

Statistical analysis

IBM SPSS Statistics for Windows, Version 25, (Released 2017; IBM Corp., Armonk, New York, United States) was used for statistical analysis using the Student’s t-test or one-way ANOVA (analysis of variance). Statistical significance was set at p < 0.05.

## Results

LIPC mRNA expression is upregulated in HNSCC tumors

**Figure 1:**
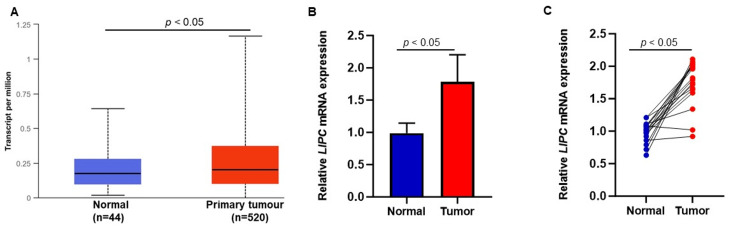
LIPC gene expression in cancer samples. (A) LIPC gene expression showed a significant difference between head and neck squamous cell carcinoma and normal samples. (B) Real-time polymerase chain reaction (qPCR) results showed that the LIPC gene was upregulated in oral squamous cell carcinoma (OSCC) samples compared with normal samples, which is similar to the UALCAN (The University of ALabama at Birmingham CANcer data analysis portal) database results. (C) LIPC gene expression in matched OSCC tumor samples and adjacent non-tumor samples from the same patient.

In our comprehensive analysis, we delved into the expression of the LIPC gene (mRNA) in both tumor and normal samples. To ensure a robust examination, we employed a dual approach utilizing qPCR and the UALCAN database. The UALCAN database revealed compelling results, indicating a significant overexpression of the LIPC gene in HNSCC compared to normal samples (Figure 1A, p < 0.05). In concordance with these findings, our qPCR results further underscored the substantial correlation between LIPC overexpression and OSCC (Figure 1B, p < 0.05). Employing the same set of samples obtained from patient tumors and control samples, our investigation extended to highlight the evident overexpression of LIPC in OSCC tumor samples when juxtaposed with adjacent normal samples (Figure 1C, p < 0.05). These findings collectively provide robust evidence supporting the notion that LIPC gene overexpression is a notable characteristic in both HNSCC and OSCC, as substantiated by both the UALCAN database and our independent qPCR analyses.

Clinicopathological features of HNSCC correlated with LIPC gene expression

**Figure 2:**
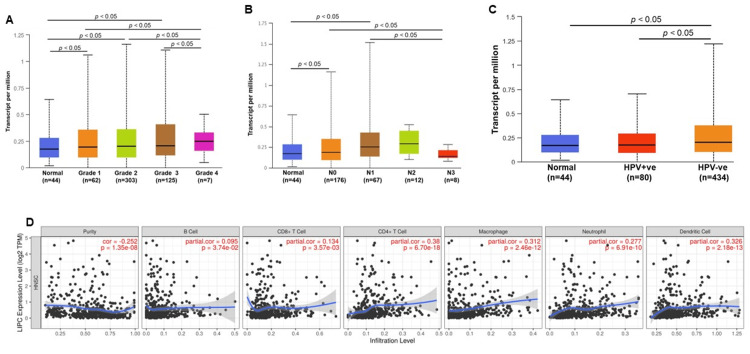
Clinicopathological features of LIPC in head and neck squamous cell carcinoma (HNSCC). LIPC was significantly correlated with clinicopathological features of HNSCC. (A) In tumor grade, HNSCC gene expression significantly overexpressed compared with normal samples. (B) Based on the nodal metastasis status, the LIPC gene significantly overexpressed in N2 and N1 stage compared with normal samples. (C) Compared with normal samples, the LIPC gene highly expressed in HPV-negative HNSCC. * p < 0.05. (D) Tumor immune infiltration level results show significant results for purity, B cells, CD4+ T cells, CD8+ T cells, macrophages, neutrophils, and dendritic cells.

The exploration of clinicopathological features in HNSC) pertaining to LIPC was conducted through the UALCAN database, unraveling compelling insights. The results illuminated a significant correlation between LIPC gene expression and key clinicopathological features in HNSCC, specifically tumor grade, nodal metastasis status, and HPV status (Figure 2A-2C, p < 0.05). These findings underscore the pivotal role of LIPC in influencing the clinical characteristics of HNSCC, indicating its involvement in tumor grade determination and contributing to the metastatic potential of HNSCC. Moreover, an intriguing facet emerged from our investigation into the relationship between LIPC gene expression and immune cell infiltration in HNSCC, employing the TIMER database. The analysis showcased a robust correlation between LIPC expression and the infiltration levels of various immune cells, including B cells, CD8+ cells, CD4+ cells, macrophages, neutrophils, and dendritic cells (Figure 2D, p< 0.05). This suggests that LIPC may play a crucial role in modulating the tumor microenvironment by influencing immune cell infiltration in HNSCC. An additional noteworthy discovery from our study is the differential expression of LIPC in HPV-negative and HPV-positive HNSCC. LIPC exhibited higher expression in HPV-negative HNSCC compared to HPV-positive counterparts, implying its potential utility as a biomarker specifically for HPV-negative HNSCC (p < 0.05). These multifaceted insights shed light not only on the clinical implications of LIPC in HNSCC but also on its intricate involvement in the interplay between tumor biology and the immune microenvironment.

Prognostic and functional analysis of LIPC in HNSCC

**Figure 3:**
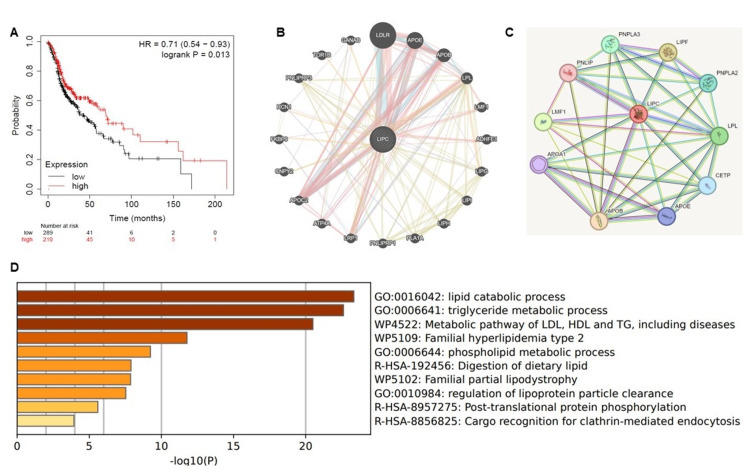
(A) Kaplan-Meier plot showing the survival rate of head and neck squamous cell carcinoma (HNSCC) related to the LIPC gene expression. The red and black color lines indicate the high and low expressions, respectively, of the LIPC gene related with survival rate. (B) GeneMANIA results show LIPC gene interaction with other genes and (C) STRING database shows LIPC protein interaction with other proteins (D) Metascape results explain the functional enrichment analysis of the LIPC gene, which is associated with lipid catabolic process, triglyceride metabolic process, and post-translational protein phosphorylation.

Kaplan-Meier plots were used to analyze HNSCC prognosis. High expression of the LIPC gene was significantly correlated with a better prognosis in patients with HNSCC (Figure 3A, p < 0.05). Gene and protein interactions were analyzed using the GeneMANIA and STRING databases. GeneMANIA provided details about LIPC interactions with other genes, such as LPL, APOB, LMF1, LIPG, LIP1, LIPH, and PLAIA (Figure 3B), as well as protein-protein interactions analyzed using the STRING database. PNPLA2, LPL, CETP, LMF1, and LIPF proteins were mainly associated with LIPC (Figure 3C). Metascape analysis revealed that the LIPC gene was involved in pathways such as lipid catabolic process, phospholipid metabolic process, triglyceride metabolic process, and post-transitional protein phosphorylation (Figure 3D).

## Discussion

In our current investigation, we sought to unravel the intricate association between LIPC expression and HNSCC. Given the global prevalence of HNSCC, the identification of reliable biomarkers and treatment targets remains a relatively uncommon feat. Thus, elucidating the relationship between HNSCC and LIPC gene expression holds significant promise in uncovering potential avenues for targeted treatment strategies. Notably, LIPC, recognized for its role in triacylglyceride hydrolysis and the production of low-density lipoproteins by aiding the release of free fatty acids from intermediate-density lipoproteins, emerged as a focal point in our study [23].

A noteworthy precedent set by Huang et al. explored LIPC expression in gastric cancer, revealing a strong correlation between upregulated LIPC gene expression and poor survival in Borrmann type 4 gastric cancer [24]. Aligning with this, our study disclosed a significant correlation between LIPC overexpression and HNSCC. Both qPCR results and the UALCAN database underscored the marked overexpression of the LIPC gene in HNSCC samples when compared with normal samples.

Curtis et al.'s exploration of LIPC in non-small cell lung carcinoma (NSCLC) indicated an adverse correlation between increased LIPC protein expression and worse prognosis and survival rates [25]. Intriguingly, our Kaplan-Meier plot analysis for HNSCC prognosis associated with LIPC gene expression revealed a distinct pattern, with LIPC upregulation significantly correlated with a better prognosis in HNSCC patients.

Leveraging the TCGA dataset, our study extended its purview to dissect LIPC expression concerning HNSCC. The UALCAN database enriched our understanding by detailing the clinicopathological features associated with LIPC in HNSCC. Remarkably, LIPC gene expression correlated with sample type, tumor grade, metastasis status, and HPV status. Of particular note was the higher expression of LIPC in HPV-negative HNSCC compared to HPV-positive counterparts, suggesting its potential as a biomarker for the analysis of HPV-associated cancers.

The association network of LIPC with other genes and proteins unveiled intriguing connections. Our study revealed that the LIPC gene is intricately linked with LPL, APOB, LMF1, and LIPG. These associations hinted at potential implications in cancer proliferation, as exemplified by Cadenas et al.'s findings in breast tumors with strong LIPG mRNA expression correlating with shorter metastasis-free survival [26]. Moreover, the network extended to protein connections, with LIPC being associated with PNPLA2, LPL, CETP, LMF1, and LIPF proteins. The connection between LIPC and LIPF, known for its involvement in lipid metabolic processes and downregulation in gastric cancer, added another layer of complexity to the potential role of LIPC in HNSCC [27].

Metascape analysis illuminated the multifaceted involvement of the LIPC gene in various pathways, notably lipid catabolic processes, triglyceride metabolic processes, phospholipid metabolic processes, and post-translational protein phosphorylation. Given the well-established connection between altered lipid metabolism and cancer, our findings suggested a plausible role for LIPC in the metabolic reprogramming crucial for cancer development [28]. The inhibition of lipid biosynthesis can help limit the survival of cancer cells and tumor growth, whereas metabolic reprogramming of lipids can also affect other processes that are crucial for the advancement of cancer, such as endoplasmic reticulum (ER) stress and ferroptosis. [29,30]. Based on the Metascape results and other research investigations, we conclude that the LIPC gene is involved in lipid metabolic pathways, which may play a role in cancer development.

The clinical relevance of LIPC gene expression in the development of HNSCC, with a particular focus on OSCC, is a subject of interest due to the potential implications for oral health. LIPC is known to play a role in lipid metabolism, and its dysregulation has been associated with various diseases, including cancer. In the context of HNSCC, including OSCC, understanding the expression patterns and potential functions of LIPC could provide insights into its involvement in tumorigenesis, prognosis, and treatment response. This knowledge may have clinical implications for risk assessment, early detection, and the development of targeted therapies for individuals at risk or diagnosed with HNSCC, thereby contributing to advancements in oral health care.

Our study identified the overexpression of LIPC in both HNSCC and OSCC tumors, aligning with previous indications of LIPC involvement in various cancer types and its potential as a diagnostic biomarker. Despite these promising findings, our study acknowledges its limitations, including a relatively small sample size, reliance on in silico analysis, and the need for extensive in vitro and in vivo functional analyses. Future endeavors should focus on expanding sample sizes and conducting rigorous functional analyses to comprehensively understand the role of LIPC in HNSCC and its potential as a therapeutic target.

## Conclusions

This study establishes a correlation between HNSCC and the LIPC gene expression, suggesting its potential as a biomarker. The significant links between LIPC expression, clinicopathological features, and prognosis indicate its substantial role in HNSCC development. Further research is needed to comprehensively understand the precise mechanisms by which the LIPC gene contributes to HNSCC. Unraveling these mechanisms could pave the way for targeted therapeutic interventions. In summary, the study highlights the importance of LIPC as a potential diagnostic and therapeutic target in HNSCC.
